# Sterilizing Instruments

**Published:** 1904-08

**Authors:** H. A. Jelly


Sterilizing Instruments.—The sterilizing of instruments is usually a bugbear to dentists. It is claimed that some expense attends it, and also that a busy dentist cannot take the time. These reasons may be true, but the necessity for sanitary means remains the same.

As a student this principle was firmly implanted in my mind, and this simple and convenient method I have found very easy. May I suggest it to another busy man?

A ring bracket is fastened to the wall, by the side of the cabinet, in which place a heavy glass, to be three parts full of water with one teaspoonful of formaldehyd. This can be easily changed during the day, by pouring the contents into the spittoon, so that the pipes can also be cleansed at the same cost. After using instruments, place them in this solution and let them remain. This is not repulsive to the patients and impresses them with the idea that they are the subjects of cleanliness. After the patient's departure, these instruments can be used as one uses an egg-beater and thus perfectly cleansed, wiped dry, and they are ready for the next patient.

Our experience has been that this care has always been highly appreciated.—Dr. H. A. Jelly, Cosmos.

